# New alternatives to the Lennard-Jones potential

**DOI:** 10.1038/s41598-024-60835-8

**Published:** 2024-05-15

**Authors:** Pablo Moscato, Mohammad Nazmul Haque

**Affiliations:** 1 School of Information and Physical Sciences, The University of Newcastle, Callaghan, NSW 2308 Australia; 2 ResTech Pty Ltd, CE Building, Design Drive, Callaghan, NSW 2308 Australia

**Keywords:** Lennard-Jones potential, Dirichlet polynomial, Symbolic regression, Analytic continued fraction, Memetic algorithm, Computational science, Information theory and computation

## Abstract

We present a new method for approximating two-body interatomic potentials from existing *ab initio* data based on representing the unknown function as an analytic continued fraction. In this study, our method was first inspired by a representation of the unknown potential as a Dirichlet polynomial, i.e., the partial sum of some terms of a Dirichlet series. Our method allows for a close and computationally efficient approximation of the *ab initio* data for the noble gases Xenon (Xe), Krypton (Kr), Argon (Ar), and Neon (Ne), which are proportional to $r^{-6}$ and to a very simple $depth=1$ truncated continued fraction with integer coefficients and depending on $n^{-r}$ only, where *n* is a natural number (with $n=13$ for Xe, $n=16$ for Kr, $n=17$ for Ar, and $n=27$ for Neon). For Helium (He), the data is well approximated with a function having only one variable $n^{-r}$ with $n=31$ and a truncated continued fraction with $depth=2$ (i.e., the third convergent of the expansion). Also, for He, we have found an interesting $depth=0$ result, a Dirichlet polynomial of the form $k_1 \, 6^{-r} + k_2 \, 48^{-r} + k_3 \, 72^{-r}$ (with $k_1, k_2, k_3$ all integers), which provides a surprisingly good fit, not only in the attractive but also in the repulsive region. We also discuss lessons learned while facing the surprisingly challenging non-linear optimisation tasks in fitting these approximations and opportunities for parallelisation.

## Introduction

The Lennard-Jones (LJ) potential has once been considered *“one of the centerpieces in Molecular Dynamics (MD) simulations, the key computational method for studying atomistic phenomena across Chemistry, Physics, Biology, and Mechanics”*^1^. It is a well-known functional form proposed for approximating two-body interatomic potentials when existing data is available. While the LJ potential possesses favourable mathematical properties, it is a special case of a more general parameterisable functional form attributed to Mie^2^.

Despite its widespread fame and extensive use, the LJ potential may not accurately represent certain characteristics of specific physical interactions. Consequently, a model-independent mathematical method that can directly “learn from data” the specific functional form of a two-body interaction potential without making excessive assumptions is a crucial research endeavour. This is particularly relevant in symbolic regression, where developing reliable identification methods for accurate approximations of two and three-body potentials would greatly enhance data-driven model building. However, this task poses a significant challenge, even when dealing with relatively small datasets and highly non-linear target functions, as is the case here.

It is essential to acknowledge that this problem is far from being solved. The field has a rich history of a century of research, and numerous potentials, bearing the names of their proposers, are now widely used in molecular dynamics simulations. For instance, a comprehensive review of many proposed potentials can be found in Ref.^3^.

Deriving an analytical form for the potential from experimental data poses a more significant challenge for machine learning approaches. However, it remains an area of interest for testing new data-driven methods, such as those proposed in Ref.^1^, which utilise data provided by Halpern for the Argon dimer^4^. In this paper, we also revisit this dataset and utilise *ab initio* data available for Xenon (Xe), Krypton (Kr), Argon (Ar), Neon (Ne), and Helium (He) from several publications, including the work by Jäger, Hellmann, Bich, and Vogel^5^, as well as the comprehensive study by Deiters and Sadus in 2019 (see Ref.^3^ and the references cited therein).

### The Lennard-Jones potential

The Lennard-Jones potential $V_{LJ}(r; \sigma , \varepsilon , n, m)$ for a pair of interacting particles is defined by Eq. (1),

1 $$\begin{aligned} V_{LJ}(r; \sigma , \varepsilon , n, m)= 4 \varepsilon \left[ \left( \frac{\sigma }{r}\right) ^n - \left( \frac{\sigma }{r}\right) ^m\right] , \end{aligned}$$

where *r* is the distance between the interacting atoms and *n* is the ‘repulsion exponent’ and historically^1^, by mathematical convenience at the time, it was set as $n=12$, $m=6$ and the values of $\sigma$, $\varepsilon$ are chosen according to the available experimental data. Clearly the potential has a single root ($V_{LJ}(\sigma ) = 0$) and a minimum at $r_{min}= 2^{1/6} \sigma$ and $V_{LJ}(r_{min}) = -\sigma$.

### The Mie Potential and Kulakova’s approximation with non-integers *n* and *m*

It is known that Lennard-Jones explored various values for the parameters *n* and *m* before arriving at the final form. In 2017, Lina Kulakova and her colleagues conducted an intriguing study in which they investigated the joint calibration of all parameters in the Lennard-Jones functional form, allowing for non-integer values of *n* and *m*^1^. They concluded that *“the repulsion exponent* $n \approx 6.5$ *provides an excellent fit for experimental data of liquid argon across a range of thermodynamic conditions, as well as for saturated argon vapor”*. However, when using the quantum simulation data of the Argon dimer made available by Arthur M. Halpern in 2010^4^, a good fit was not obtained with $p=12$. The data suggested that values of $n \approx 12.7$ are *“preferred for Argon gas, while experimental data support lower values”*.

It is worth noting that many decades before 2017, there was a similar proposal; an even more general form of the LJ potential was proposed by the German physicist Gustave Mie in 1903^2^:

2 $$\begin{aligned} V_{Mie}(r; \sigma , \varepsilon , n, m)= \frac{n}{n-m} \left( \frac{n}{m}\right) ^{\frac{m}{n-m}} \varepsilon \left[ \left( \frac{\sigma }{r}\right) ^n - \left( \frac{\sigma }{r}\right) ^m\right] . \end{aligned}$$

The potential has a root at $r=\sigma$ and a minimum at $r_{min}$ given by

$$\begin{aligned} r_{min} = e^{f(n,m,\sigma )} \end{aligned}$$

where

$$\begin{aligned} f(n,m,\sigma ) = \frac{m\; ln(\sigma ) + ln(m) - n\; ln(\sigma ) - ln(n)}{(m - n)}. \end{aligned}$$

So for $n=12$ and $m=6$, we have that the minimum is

3 $$\begin{aligned} V_{Mie}(r_{min}; \sigma ) = 4 \, \varepsilon \, \sigma ^6 \, \left( \frac{\sigma ^6 - r_{min}^6}{r_{min}^{12}}\right) . \end{aligned}$$

It is important to remark that this functional form of the Mie potential (given by Eq. (3)), which is frequently attributed to Ref.^2^, does not appear in that manuscript. In fact, it seems that a generalised form first appeared in a textbook^6^ in 1939. We are indebted to R. Sadus, who communicated this fact to us.

### Buckingham and other proposed potentials

In 1938, while studying the equation of state for gaseous helium, neon and argon, Richard Buckingham proposed a simplification of the Lennard-Jones potential^7^

4 $$\begin{aligned} V_{Buck}(r; A, B, C) = A \, e^{-B \, r} - \frac{C}{r^6}, \end{aligned}$$

where *A*, *B*, and *C* are constants. It is important to note that this functional form has a caveat. As the interatomic distance *r* approaches zero, the first term tends to a constant value, while the second term diverges and becomes negative for small *r*, indicating an attractive force. Consequently, it loses its physical relevance for very close interatomic distances. This problem is not present in both the Mie and Lennard-Jones potentials. We highlight this fact because, throughout the 20th century, introducing problem domain (i.e., physical) information has been crucial in the proposal of several alternative functional forms. We will discuss this issue in the ‘Introducing problem domain information’ section.

Other recently proposed functional forms of interest have been extensively discussed in Ref.^3^, so we refer the reader to that paper for more information on these potentials.

In the same paper^3^, Deiters and Sadus introduced a general functional form for a potential called SAAPx, which requires fixing seven coefficients for Helium (He) and six coefficients for the other noble gases Xenon (Xe), Krypton (Kr), Argon (Ar), and Neon (Ne).

## Analytic continued fractions and symbolic regression methods

We will start with a simple introduction to symbolic regression to understand how our proposal was data-driven.

### An example of symbolic regression

To illustrate how symbolic regression works, let’s assume we are given values of an unknown function *f*(*r*) on some points (i.e. no experimental error in this case) so we know that the values in the given set $\{(r,f(r))\}$ are perfectly known. See, for instance, Table 1 as an illustrative example.

**Table 1:** An example of a hypothetical function *f*(*r*) to be learned from the existing data with *r* a positive integer $1 \le r \le 9$.

*r*	1	2	3	4	5	6	7	8	9
*f*(*r*)	1	2	4	8	16	31	57	99	163

The unknown function in this case relates to *Moser’s circle problem*, and further details are given in Supplementary Material’s Appendix 3. This example is used here to illustrate the discussion on symbolic regression approaches.

Current symbolic regression methods, such as the one implemented at the core of the TuringBot software, have demonstrated remarkable power (see the methods used in Ref.^8^ and their results on a large variety of datasets). They are capable of “learning” from data using a number of built-in mathematical functions.

For this illustrative task, we have employed the TuringBot software (which implements a symbolic regression approach) to obtain the following function:

5 $$\begin{aligned} f_1(r)=Round\left( \left( r - sinh\left( cos\left( \sqrt{r}\right) \right) \right) ^{ln(r)}\right) \end{aligned}$$

which does not make any error in the training data and “predicts” that $f_1(10)=259$, $f_1(11) = 399$ and $f_1(12)=597$.

We have obtained a formula with no coefficients and no error in the training data. This may be appealing, but we may suspect that this formula may not be the “true unknown function” we are trying to approximate. To deal with this, TuringBot, like many other symbolic regression packages, allows you to “unselect” many mathematical functions used as “building blocks” provided as default. In fact, we could search for functions using “just” integer coefficients and only the basic arithmetic functions of addition, subtraction, multiplication and division.

In this case, we have been able to use symbolic regression solvers to obtain, for instance, a simple polynomial equation in $u=r-1$ such as:

6 $$\begin{aligned} f_{g.t} (r) = \frac{24 + 14 u + 11 u^2 - 2 u^3 + u^4}{24} \end{aligned}$$

which also perfectly fits the data and for which $f_{g.t}(10)=256$, $f_{g.t}(11) = 386$ and $f_{g.t}(12)=562$, which, as perhaps expected, do not agree with those of Eq. (5). We can rewrite it as:

7 $$\begin{aligned} f_{g.t} (r)=\frac{r^4 - 6 r^3 + 23 r^2 - 18 r + 24}{24}. \end{aligned}$$

for any integer $r \ge 1$.

Without further addition of problem domain knowledge about the nature of the unknown function *f*(*r*), both Eqs. (5) and  (7) can equally be the function (as well as infinitely many others that fit the training data).

We will return to this motivating example later. Still, at this point, we want to remark that we can think of Eq. (7) as an approximation using ratios of polynomials in *r* with integer coefficients. When searching for relatively simpler equations, it is frequently the case that a change of variables may help to reduce the complexity of the final model. This simple illustration paves the way for discussing the following topics since we propose a novel representation.

### Analytic continued fraction regression

Since 2019 we have been championing a new approach for multivariate regression. It is based on representing the unknown target function as an analytic continued fraction. The resulting method, called Continued Fraction Regression (cfr), has been demonstrated to have competitive performance on a variety of regression problems^9,10^, including in materials science^11^ and physics^12,13^. In Ref.^14^, using 352 datasets from real experiments in the physical and chemical sciences, CFR showed, employing leave-one-out cross-validation, that it was ranked first in 350 out of the 352 datasets (in training) in comparison with ten machine learning regression methods of the scikit-learn collection. In testing, CFR ranked first 192 times, i.e. more than all of the other ten algorithms combined.

In CFR, it is proposed that the target function of a multivariate regression problem can be represented as an analytic continued fraction. For a multivariate input $\textbf{x}=[x_1,x_2,\cdots ,x_d]$, where *d* is the number of variables, an output $y \in \mathbb {R}$, a regression model is defined as an analytic continued function ${f}:\mathbb {X} \rightarrow \mathbb {Y}$ with $\mathbb {Y} \subseteq \mathbb {R}$. In Ref.^10^ Eq. (8) was first proposed, as a first approach to developing the theory, to start studying the representation potential of this functional form:

8 $$\begin{aligned} {\begin{matrix} f(\textbf{x}) = g_0(\textbf{x})+ \frac{{h_0(\textbf{x})}}{{g_1(\textbf{x})}+ \frac{{h_1(\textbf{x})}}{{g_2(\textbf{x})}+ \frac{{h_2(\textbf{x})}}{{g_3(\textbf{x})}+ \genfrac{}{}{0.0pt}0{}{{\ddots }+ \frac{{h_n(\textbf{x})}}{{g_n(\textbf{x})}}}}}} \end{matrix}} \end{aligned}$$

In Eq. (8) for a continued fraction with depth *n*, we have $g_i(\varvec{x}) \in \mathbb R$ for all integers *i* such that $0 \le i \le n$. Each $g_i:\mathbb {R}^n \rightarrow \mathbb {R}$ is associated with an array $\varvec{a}_i\in \mathbb {R}^n$ and a constant $\alpha _i \in \mathbb {R}$. Analogously, each $h_i:\mathbb {R}^n \rightarrow \mathbb {R}$ is defined by an array $\varvec{b}_i\in \mathbb {R}^n$ and a constant $\beta _i \in \mathbb {R}$. We thus define:

9 $$\begin{aligned} g_i(\textbf{x}) = \textbf{a}_i^T \textbf{x}+\alpha _i, \end{aligned}$$

10 $$\begin{aligned} h_i(\textbf{x}) = \textbf{b}_i^T \textbf{x}+\beta _i. \end{aligned}$$

We note that the full representational power behind CFR is more general, and other functional forms for the functions $g_i$ and $h_i$ can be used. It has been a conscious design choice to start exploring the power of this representation by restricting these base functions to be linear. We refer to Ref.^9^ to see how complex functions, like the Gamma Function, can be well approximated using these choices and how they perform on 94 real-world datasets of the Penn Machine Learning Database.

### A Dirichlet-inspired representation

A *general Dirichlet series* is an infinite series of the form

11 $$\begin{aligned} \sum _{n=1}^{\infty } a_n e^{-\lambda _n \, s}, \end{aligned}$$

where $a_n$ and *s* are complex numbers and the set $\{\lambda _n\}$ is a strictly increasing sequence of non-negative real numbers that tend to infinity. When $\lambda _n = ln(n)$ we have the *“ordinary” Dirichlet series*. One of the most famous of them is the Riemann zeta function which has applications in physics, statistics and many branches of mathematics and is defined as

12 $$\begin{aligned} \zeta (s) = \sum _{n=1}^{\infty } \frac{1}{n^s} \end{aligned}$$

where $Re(s)>1$ and its analytic continuation elsewhere.

This has suggested a new representation; for a large value of an integer *N*, it may be possible to approximate the potential value between two molecules (labelled 1 and 2) at a distance *r*. We can write

13 $$\begin{aligned} V_{1,2}(r) = \sum _{n=1}^{N} \frac{a_n}{n^r} \end{aligned}$$

so the problem of finding the best approximation for $V_{1,2}$ has now reduced to the problem of finding the set $\{a_1, a_2, \dots , a_N\}$.

In Supplementary Material’s Appendix 1, we show how to use symbolic regression software to search for continued fraction approximations of an unknown potential using the Dirichlet representation. We illustrate the methods using Halpern’s Argon dataset.

## Introducing problem domain information

In this section, we show how we can get very good solutions for several Noble gases using the same dataset employed by Deiters and Sadus^3^. It is worth mentioning that all potential values $V_{1,2}(r)$ are dimensionless, and the variable *r* is measured in nanometers (nm) in this work.

### Deiters and Sadus’s SAAP two-body potential and the introduction of problem-domain information

In 2019, Deiters and Sadus presented a two-body potential for the noble gases Ne, Ar, Kr, and Xe, which is called SAAP, an acronym for *‘Simplified Ab initio Atomic Potential* (and a variant of it called SAAPx for Helium)^3^. They provided a set of rules originating from their physical understanding of the problem domain that can help design a useful functional form that fits experimental data for all these gases well. First of all, the asymptotic behaviours that are desired should be taken into consideration:

- It is known that $V_{1,2}(r)$ should be approaching zero for large values of *r* as a function of $r^{-6}$ (and have negative values). They validate their claim by saying that dispersion interactions dominate the potential; this means that the original Lennard-Jones potential had that specific asymptotic behaviour already “hardwired” in the functional form.
- When *r* tends to zero, there is a repulsion effect (Pauli repulsion), and $lim_{r \rightarrow 0} V_{1,2}(r) = \infty$.

They also propose the following behaviour for the potential (our rephrasing):

- Following the same Mie potential convention, let $\sigma ~>~0$ be the value satisfying $V_{1,2}(\sigma ) = 0$. Such a value is unique for all $r>0$ and in addition $dV/dr < 0$ for all $0 < r \le \sigma$ (and it is called the “collision diameter”).

Deiters and Sadus include another source of problem domain information. In two previous articles by Pathak and Thakkar^15^ as well and by Deiters and Neumaier^16^, an expression of the form *exp*(*r*)/*r* was proposed for the repulsion. Then the proposed formula for SAAP is:

14 $$\begin{aligned} V_{1,2}(r) \approx SAAP(r) = \frac{\frac{a_0}{r} e^{a_1 \, r} + a_2 \, e^{a_3 \, r } + a_4 }{1+ a_5 \, r^6} \end{aligned}$$

where $a_1, \dots , a_4 < 0$ and $a_0, a_5 > 0$. These six coefficients are then to be adjusted using the experimental data.

Remembering then the definition of a general Dirichlet series, it is then interesting to note that SAAP resembles a two-body potential of the form:

15 $$\begin{aligned} V_{1,2}(r) \approx \frac{\sum _{n=1}^{\infty } b_n \, e^{-\lambda _n \, r}}{1+r^6}. \end{aligned}$$

### New fits with continued fraction regression with asymptotic behaviour as $r^{-6}$

Following Deiters and Sadus’s approach of introducing problem domain information, we now propose to approximate $V_{1,2}(r)$ as:

16 $$\begin{aligned} V_{1,2}(r) = \frac{1}{r^6} \sum _{n=1}^{N} \frac{a_n}{n^r}, \end{aligned}$$

so, in this case, we would multiply the value of the observed values at any given *r* by $r^6$ to find truncated continued fraction approximations. Interestingly, for Xenon, Krypton, Argon and Neon, we obtained.

#### Xenon

17 $$\begin{aligned} V_{1,2}(r) = r^{-6} \, \left( - 2975348 + \frac{80437659232 \times 13^{-r}}{4735 \times 13^{-r} + 1} \right) \end{aligned}$$

with $$\sigma \approx 3.90352$$ and $$r_{min} \approx 4.34565$$,

with $$V_{1,2}(r_{min}) \approx -280.4872$$.

#### Krypton

18 $$\begin{aligned} V_{1,2}(r) = r^{-6} \, \left( - 1270942 + \frac{30538104125 \times 16^{-r}}{2961 \times 16^{-r} + 1} \right) \end{aligned}$$

with $$\sigma \approx 3.59067$$

and $$r_{min} \approx 3.98787$$,

with $$V_{1,2}(r_{min}) \approx -201.5254$$.

#### Argon

19 $$\begin{aligned} V_{1,2}(r) \approx r^{-6} \, \left( -641200 + \frac{10640800000 \times 17^{-r}}{2728 \times 17^{-r} + 1 } \right) \end{aligned}$$

with $\sigma \approx 3.36624$ and $r_{min} \approx 3.7528$, with $V_{1,2}(r_{min}) \approx -143.335$.

#### Neon

20 $$\begin{aligned} V_{1,2}(r) = r^{-6} \, \left( - 58578 + \frac{603430988 \times 27^{-r}}{1138 \times 27^{-r} + 1} \right) \end{aligned}$$

with $\sigma \approx 2.76803$ and $r_{min} \approx 3.08297$, with $V_{1,2}(r_{min}) \approx -42.20354$. We highlight that all these formulas can be rearranged in the form $V_{1,2}(r) \approx r^{-6} \, (a_0 + a_1/(a_2 + n^r))$ that requires a single computation of an exponential (to the power of *r* only) and only have four free adjustable integer coefficients. We believe that these relatively simpler forms have the potential to lead towards more efficient molecular dynamics simulations.

#### SAAPx and our model for Helium

To fit the *ab initio* data from Helium, Deiters and Sadus proposed a modification of the SAAP potential and called it *SAAPx*. It needs an extra coefficient to be empirically fitted from the data ($a_6$). Formally it is written as:

21 $$\begin{aligned} V_{1,2}(r) \approx SAAP_x(r) = \frac{\frac{a_0}{r} e^{a_1 \, r + a_6 \, r^2} + a_2 \, e^{a_3 \, r } + a_4 }{1+ a_5 \, r^6} \end{aligned}$$

Clearly, $SAAP_x(r)$, can be seen as just a generalisation of *SAAP*(*r*), so it is reasonable to state that Deiters and Sadus’s proposal for these potentials is based on a functional form with *seven* adjustable parameters, with $a_6$ being ad hoc set to zero for all other noble gases that are not Helium.

In contrast, we continue with our investigation of the representational properties derived from our proposal of eq. (16). We will present two functions that we have found that fit the experimental data relatively well.

22 $$\begin{aligned} V_{1,2}(r) = 6^{-r} \left( 4521391 \times 12^{-r} - 645460 \times 8^{-r} - 3732 \right) \end{aligned}$$

with $\sigma \approx 2.64036$ and $r_{min} \approx 2.97924$, with $V_{1,2}(r_{min}) \approx -11.01906$.

23 $$\begin{aligned} V_{1,2}(r) = r^{-6} \times \left( \frac{29173.2876433231}{ \left( 14.2052906553669\times 31^{-r} + 0.348000451488318 \right) + \frac{0.000325594052656555}{31^{-r} -0.000114634476140062} } \right) \end{aligned}$$

We have found an approximation of the model in Eq. (23) as a simplified format as follows:

24 $$\begin{aligned} V_{1,2}(r) = r^{-6} \times \left( \frac{-11973656257\times 31^r }{5000000\times 31^r+6807301800} \right) \times \left( \frac{500000\times 31^r-4503812769 }{100000\times 31^r+4019217} \right) \end{aligned}$$

with $\sigma \approx 2.65168$ and $r_{min} \approx 2.9572$, with $V_{1,2}(r_{min}) \approx -11.03116$.

**Figure 1:**
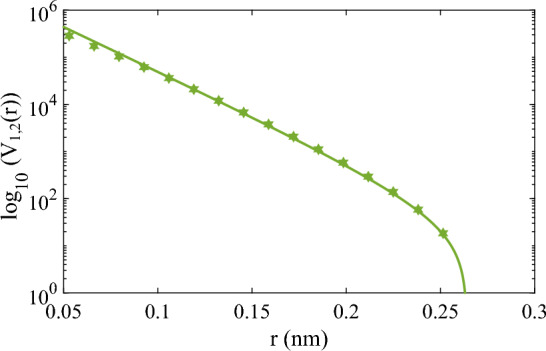
Comparison of the *ab initio* potential energy of He (green solid stars) with our model’s (the solid green line with Eq. (22)) calculations at interatomic separations in the repulsive region.

**Figure 2:**
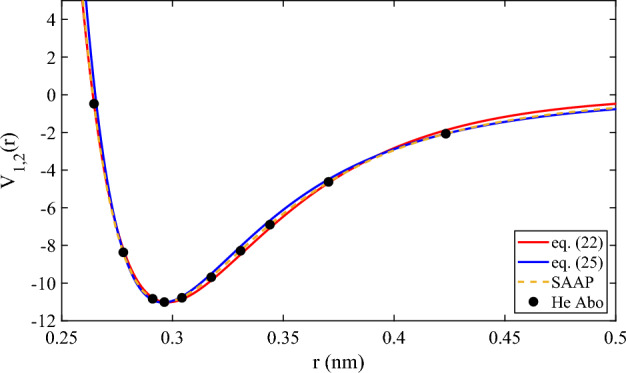
Comparison of the *ab initio* potential energy of He (black solid dots) with SAAP (orange dashed line) and our model’s (the solid red for Eq. (22) and blue for Eq. (25)) calculations at interatomic separations close to the attractive well.

Figure 1 shows the comparison of *ab initio* potential energy of He and approximation by Eq. (22) at interatomic separations in the repulsive region and Fig. 2 shows the comparison of *ab initio*, SAAP and approximation by Eq. (25) close to the attractive well.

#### Plots for all $depth=1$ or $depth=2$ (He) models

**Figure 3:**
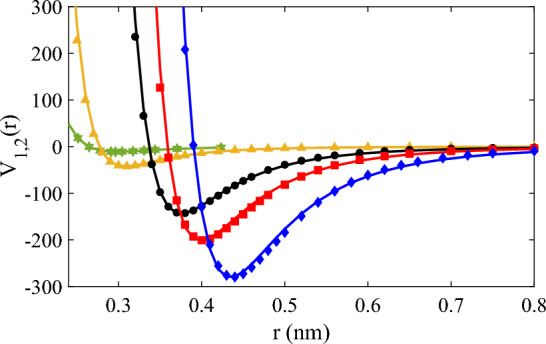
Comparison of *ab initio* potential energies with corresponding model calculations (solid lines) for He (green solid stars) with Eq. (22), Ne (orange solid triangles) with Eq. (20), Ar (black solid circles) with Eq. (19), Kr (red solid squares) with Eq. (18), and Xe (blue solid diamonds) with Eq. (17) at interatomic separations close to the attractive well.

We show the comparison of ab inito potential energies along with the corresponding models for He, Ne, Ar, Kr and Xe gases in Fig. 3. We have computed the relative error (RE) to assess the fitting of data points by respective models, and the results are summarised in Table 2 for a more accessible and concise presentation of our findings.

**Table 2:** Comparison of relative error (RE) for predicting the *ab initio* potential energies for He by eq. (22), Ne by eq. (20), Ar by eq. (19), Kr by with eq. (18) and Xe by eq. (17).

He			Ne			Ar			Kr			Xe		
r	$V_{1,2}( r )$	RE	r	$V_{1,2}( r )$	RE	r	$V_{1,2}( r )$	RE	r	$V_{1,2}( r )$	RE	r	$V_{1,2}( r )$	RE
0.052918	286570	0.2570	0.14	68634.3	0.2061	0.18	98948.5	0.1077	0.22	53147.5	0.2167	0.24	67031.2	0.0262
0.066147	173854	0.1955	0.16	26879.9	0.1443	0.2	51406.2	0.1401	0.24	27872.3	0.2281	0.26	37578.5	0.0016
0.079377	104343	0.1371	0.18	10402.2	0.0415	0.22	25736.3	0.1029	0.26	14136.1	0.2485	0.28	20522.1	0.0435
0.092606	61787.5	0.0852	0.2	3918.98	0.0522	0.24	12404.6	0.0335	0.28	6856.11	0.2619	0.3	10810.4	0.0949
0.105835	36150.5	0.0396	0.22	1399.23	0.1103	0.26	5701.43	0.0364	0.3	3106.29	0.2591	0.32	5404.88	0.1364
0.119065	20911.2	0.0003	0.24	444.443	0.1302	0.28	2439.02	0.0874	0.32	1242.57	0.2397	0.34	2481.02	0.1555
0.132294	11961.9	0.0330	0.25	227.49	0.1311	0.3	912.136	0.1150	0.33	716.617	0.2272	0.35	1592.04	0.1550
0.145524	6760	0.0598	0.26	99.956	0.1336	0.31	502.756	0.1236	0.34	361.343	0.2192	0.36	958.642	0.1487
0.158753	3768.15	0.0798	0.27	27.186	0.1734	0.32	235.724	0.1371	0.35	126.701	0.2430	0.37	514.265	0.1383
0.171983	2066.63	0.0932	0.28	− 12.388	0.1328	0.33	66.058	0.2025	0.36	− 23.509	0.7933	0.38	208.418	0.1292
0.185212	1110.65	0.0998	0.29	− 32.165	0.0005	0.34	− 37.753	0.1754	0.37	− 115.326	0.0172	0.39	3.312	0.9064
0.198441	581.162	0.1004	0.3	− 40.392	0.0051	0.35	− 97.643	0.0070	0.38	− 167.342	0.0288	0.4	− 129.334	0.0562
0.211671	292.634	0.0958	0.31	− 42.13	0.0005	0.36	− 128.726	0.0062	0.39	− 192.735	0.0181	0.41	− 210.472	0.0442
0.2249	138.517	0.0864	0.32	− 40.423	0.0052	0.37	− 141.366	0.0044	0.4	− 200.741	0.0035	0.42	− 255.56	0.0276
0.23813	58.4111	0.0738	0.33	− 37.04	0.0089	0.38	− 142.546	0.0007	0.41	− 197.781	0.0107	0.43	− 275.892	0.0113
0.251359	18.3547	0.0599	0.34	− 33.032	0.0105	0.39	− 136.953	0.0059	0.42	− 188.246	0.0230	0.44	− 279.634	0.0033
0.264589	− 0.4774	0.0384	0.35	− 28.972	0.0098	0.4	− 127.658	0.0098	0.43	− 175.11	0.0326	0.45	− 272.638	0.0156
0.277818	− 8.3667	0.0198	0.36	− 25.137	0.0064	0.41	− 116.63	0.0118	0.44	− 160.33	0.0392	0.46	− 259.034	0.0254
0.291047	− 10.8336	0.0056	0.37	− 21.678	0.0014	0.42	− 105.114	0.0118	0.45	− 145.167	0.0430	0.47	− 241.706	0.0327
0.296339	− 11.0085	0.0001	0.38	− 18.629	0.0053	0.43	− 93.849	0.0098	0.46	− 130.404	0.0440	0.48	− 222.63	0.0374
0.304277	− 10.7796	0.0074	0.4	− 13.723	0.0215	0.44	− 83.251	0.0058	0.47	− 116.498	0.0426	0.49	− 203.141	0.0398
0.317506	− 9.6869	0.0176	0.42	− 10.136	0.0406	0.45	− 73.536	0.0001	0.48	− 103.683	0.0390	0.5	− 184.042	0.0397
0.330736	− 8.2821	0.0237	0.44	− 7.554	0.0594	0.46	− 64.786	0.0069	0.5	− 81.609	0.0268	0.52	− 149.04	0.0339
0.343965	− 6.8976	0.0251	0.46	− 5.69	0.0772	0.48	− 50.128	0.0238	0.52	− 64.073	0.0100	0.54	− 119.495	0.0219
0.370424	− 4.6257	0.0112	0.48	− 4.34	0.0926	0.5	− 38.825	0.0427	0.54	− 50.404	0.0092	0.56	− 95.464	0.0052
0.423342	− 2.0684	0.0987	0.5	− 3.345	0.1071	0.52	− 30.207	0.0624	0.56	− 39.836	0.0291	0.58	− 76.301	0.0148
			0.52	− 2.605	0.1205	0.54	− 23.66	0.0816	0.59	− 28.321	0.0583	0.6	− 61.18	0.0365
			0.56	− 1.631	0.1412	0.56	− 18.674	0.0999	0.62	− 20.458	0.0849	0.62	− 49.288	0.0592
			0.6	− 1.058	0.1573	0.59	− 13.298	0.1244	0.65	− 15.023	0.1082	0.64	− 39.932	0.0821
			0.65	− 0.643	0.1721	0.62	− 9.643	0.1454	0.7	− 9.294	0.1396	0.66	− 32.569	0.1039
			0.7	− 0.406	0.1846	0.65	− 7.113	0.1632	0.75	− 5.97	0.1640	0.69	− 24.262	0.1357
			0.8	− 0.179	0.1990	0.7	− 4.432	0.1868	0.8	− 3.96	0.1832	0.72	− 18.336	0.1645
						0.75	− 2.865	0.2048	0.9	− 1.889	0.2101	0.75	− 14.034	0.1911
						0.8	− 1.911	0.2187	1	− 0.982	0.2273	0.8	− 9.223	0.2306
						0.9	− 0.918	0.2391	1.2	− 0.319	0.2505	0.85	− 6.248	0.2626
						1	− 0.479	0.2530	1.5	− 0.082	0.2651	0.9	− 4.343	0.2891
						1.2	− 0.156	0.2735				1	− 2.237	0.3301
						1.5	− 0.04	0.2894				1.2	− 0.724	0.3763
												1.5	− 0.184	0.4196

## Discussion

It is perhaps proper to highlight again that the choice of a good representation governs the process of finding approximations of potentials and the many aspects involved in obtaining a good fit via a computer-based optimisation process. We thus consider that there is merit in continuing to investigate how to improve these fits, using these functional forms, perhaps with more powerful optimisation approaches than the ones we have used so far. For instance, in regards to Eq. (22), we have also found another similar equation with different values for the integers of the associated Dirichlet polynomial:

25 $$\begin{aligned} V_{1,2}(r) = 6^{-r} \; \left( 4021904\times 10^{-r} - 670763\times 6^{-r} - 3287 \right) \end{aligned}$$

with $\sigma \approx 2.6407$ and $r_{min} \approx 2.9706$, with $V_{1,2}(r_{min}) \approx -11.01307$.

**Figure 4:**
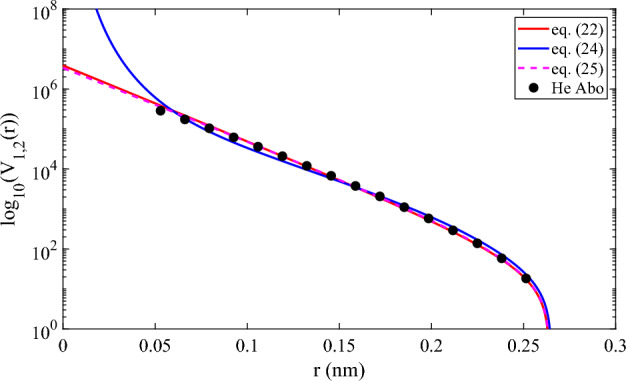
Comparison of the *ab initio* potential energy of He (black solid circles) with our model’s (the solid red for Eq. (22), solid blue for Eq. (24) and dashed magenta for Eq. (25) calculations st interatomic separations in the repulsive region where the potential takes positive values.

Figure 4 shows the comparison of the approximations of Eqs. (22), (24) and (25), in the range of the repulsive region where the potential is positive. We can see the effect of the introduction of problem-domain knowledge. In the case of Eq. (24), the $depth=2$ truncated continued fraction now has an asymptotic behaviour which is very different from data-driven generated equations Eqs. (24) and (25). However, in this range of values of *r*, for which *ab initio* data was used to fit parameters, the approximations given by the Dirichlet polynomials were very good. In fact, Eqs. (22) and (25) were in some practical sense “easier” to fit than the $depth=2$ Eq. (24). We will return to this issue later when we discuss the optimisation lessons learned in the process. We should also note that in Fig. 2, we have plotted the results of four equations, and the results of SAAP (which, surprisingly, seems to be even better than the Helium-ad hoc potential SAAPx in this region, see Fig. 3 of Ref.^3^), so it is clear that, near the minimum, these are also good approximations.

**Figure 5:**
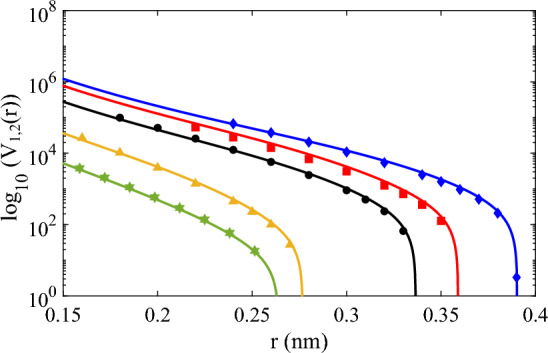
Comparison of the *ab initio* potential energy for Ar (black solid circles), He (green solid stars), Ne (orange solid triangles), Kr (red solid squares), and Xe (blue solid diamonds) with corresponding model calculations of solid lines (He (green) with Eq. (22), Ne (orange) with Eq. (20), Ar (black) with Eq. (19), Kr (red) with Eq. (18), and Xe (blue) with Eq. (17)).

We show the comparison of the *ab initio* potential energy and approximation of the models for all gases in the range of $r=\{0.15,\cdots ,0.4\}$ in Fig. 5 in log scale.

## Conclusions

This study emphasises the challenges in deriving analytical potentials from experimental data, especially when using machine learning approaches. This challenge is particularly significant in the context of accurately modelling two-body interaction potentials without making excessive assumptions. Problem-domain knowledge about asymptotic behaviour, together with a novel representation inspired by a Dirichlet series, has been an effective combined approach.

For the area of symbolic regression, the paper underscores the importance of pursuing model-independent mathematical methods that can learn the specific functional form of two-body interaction potentials directly from data. Such approaches are critical for improving data-driven model building and could be used as benchmarks for symbolic regression solvers.

The study leverages a variety of data sources, including *ab initio* data for noble gases such as Xenon (Xe), Krypton (Kr), Argon (Ar), Neon (Ne), and Helium (He). These sources also include publications by J”ager, Hellmann, Bich, and Vogel, as well as the comprehensive study by Deiters and Sadus in 2019. These data sources are invaluable for developing and testing new data-driven methods.

The research problem of deriving accurate analytical forms of interatomic potentials from data remains open and continues to be a topic of ongoing investigation. This work represents a step in that direction and highlights the need for further research in this area. The approach based on continued fraction regression seems promising as iteratively increasing depth will deliver increased fitting performance^13^. However, we have illustrated in this study how a $depth=1$ truncated continued fraction with integer coefficients is already a good approximation for this case and that the final model requires a single exponent computation.

Our paper suggests that future research may inspire novel data-driven methods, potentially improving the approximation of two and three-body potentials using continued fraction regression, including the use of dynamic depth strategies^17^. It also underscores the importance of addressing the computational challenges associated with these methods, especially when dealing with small datasets and highly non-linear target functions.

### Supplementary Information

Supplementary Information.

## Data Availability

The datasets generated during and/or analysed during the current study are available from the corresponding author upon reasonable request.
