# Supplementary material for: New alternatives to the Lennard-Jones potential
Source: Sci Rep. 2024 May 15;14:11169. doi: 10.1038/s41598-024-60835-8 (PMC11096349; doi:10.1038/s41598-024-60835-8)
Supplement: Supplementary file 1 — Supplementary Information. [file 41598_2024_60835_MOESM1_ESM.pdf]

Supplementary Information: New alternatives to the Lennard-Jones potential

Pablo Moscato[1,*] and Mohammad Nazmul Haque[1,2]

[1]School of Information and Physical Sciences, The University of Newcastle, Callaghan, NSW 2308, Australia
[2]ResTech Pty Ltd, CE Building, Design Drive, Callaghan, NSW 2308, Australia
[*]Pablo.Moscato@newcastle.edu.au

List of equations in the publication which are cited in this Supplementary Material

The proposed formula for SAAP (eq. (14) in the paper) is:

$$V_{1,2}(r) \approx SAAP(r) = \frac{\frac{a_0}{r} e^{a_1 r} + a_2 e^{a_3 r} + a_4}{1 + a_5 r^6}. \tag{1}$$

We presented a model for Helium eq. (22) in paper with $\sigma \approx 2.64036$ and $r_{min} \approx 2.97924$, with $V_{1,2}(r_{min}) \approx -11.01906$ as:

$$V_{1,2}(r) = 6^{-r} \left(4521391\, 12^{-r} - 645460\, 8^{-r} - 3732\right). \tag{2}$$

A few more alternative models for Helium are presented in teh Paper. Such as in eq. (25) with $\sigma \approx 2.6407$ and $r_{min} \approx 2.9706$, with $V_{1,2}(r_{min}) \approx -11.01307$ as:

$$V_{1,2}(r) = 6^{-r} \left(4021904\, 10^{-r} - 670763\, 6^{-r} - 3287\right) \tag{3}$$

Another approximation in eq. (23) with $\sigma \approx 2.65168$ and $r_{min} \approx 2.9572$, with $V_{1,2}(r_{min}) \approx -11.03116$ as follows:

$$V_{1,2}(r) = r^{-6} \left(\frac{29173.2876433231}{(14.2052906553669\, 31^{-r} + 0.348000451488318) + \frac{0.000325594052656555}{31^{-r} - 0.000114634476140062}} \right), \tag{4}$$

and it's simplified approximation in eq. (24) as follows:

$$V_{1,2}(r) = r^{-6} \left(\frac{-11973656257\, 31^r}{5000000\, 31^r + 6807301800} \right) \times \left(\frac{500000\, 31^r - 4503812769}{100000\, 31^r + 4019217} \right). \tag{5}$$

We have presented a simple polynomial equation (eq. (6)) in $u = r - 1$ such as:

$$f_{g.t}(r) = \frac{24 + 14u + 11u^2 - 2u^3 + u^4}{24} \tag{6}$$

which can be rewritten (in eq. (7) as:

$$f_{g.t}(r) = \frac{r^4 - 6r^3 + 23r^2 - 18r + 24}{24}. \tag{7}$$

for any integer $r \geq 1$.

Appendix 1 - Searching continued fractions using a symbolic regression software using the Dirichlet representation. An illustration using Halpern's Argon dataset.

Here we show how we can approximate an unknown potential employing a symbolic regression software and the Dirichlet representation. We start first by defining a value of N and using the set $\{1, 2^{-r}, 3^{-r}, \ldots, N^{-r}\}$ as the N-variables which the symbolic regression software will use to fit the data. This means that to model $V_{1,2}(r)$ would then to select a relatively

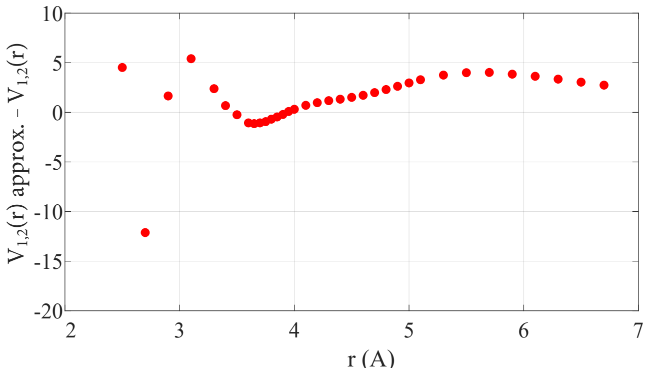

Figure 1. Error made for prediction $(V_{1,2}(r) approx - 69470\,(7^r - 714)\,34^{-r})$ of the experimental $(V_{1,2}(r))$ Argon data by eq. (8).

large integer number N, and, for each i for which we have a data in r_i generate a set of values of dependent variables $\{1, 2^{-r_i}, 3^{-r_i}, \ldots, N^{-r_i}\}$.

We can then use a symbolic regression package to find formulas with good fit and low model complexity. It is, however, entirely possible that the search would finally produce terms of the form $k\, n_1^s\, n_2^s$, with k being a constant which could be either chosen to be a real value or an integer. However, a symbolic computation package like Mathematica or Sage, can further reduce the complexity of the formula by iteratively applying the rule $n_1^{-r} n_2^{-r} = (n_1\, n_2)^{-r}$.

This is the approach we have taken here, using the TuringBot software, only allowing formulas that have integers as coefficients, followed by the use of Mathematica to further optimise the result, we have found the following approximation for Halpern's Argon dataset:

$$V_{1,2}(r) \approx -69470\,(7^r - 714)\,34^{-r} \tag{8}$$

The Mean Squared Error (MSE) of eq. (8) is 10.24589375. This could potentially be a useful approximation, when r tends to zero since it converges to a finite but large integer (49532110) and it converges to zero for r going to infinity and it has a single minimum for $r_{min} \approx 3.7892$ with $(V_{1,2}(r_{min}) \approx -96.1081)$. We also have $V_{1,2}(r) = 0$ when

$$r = 1 + \frac{ln(2) + ln(3) + log(17)}{ln(7)} \approx 3.37677. \tag{9}$$

The plot of error is shown in Fig. 1.

This is not the only simple approximation that we have found of this type, we also found two others worth mentioning:

$$V_{1,2}(r) \approx 46598041\,33^{-r} - 79879\,5^{-r} \tag{10}$$

that has a root in $r \approx 3.37496...$ and $r_{min} \approx 3.78612...$, with $(V_{1,2}(r_{min}) \approx -97.3192369...)$. This approximation has $MSE \approx 9.017775887$ in the whole dataset of 35 samples. The plot of error is shown in Fig. 2.

And we have also found one with three terms

$$V_{1,2}(r) \approx 47758692\,33^{-r} - 136107\,6^{-r} - 371\,2^{-r} \tag{11}$$

that has a root in $r \approx 3.37587...$ and $r_{min} \approx 3.79137...$, with $(V_{1,2}(r_{min}) \approx -95.896)$. This approximation has $MSE \approx 8.5568262$ so it is only slightly better than the two term approximation. The plot of error is shown in Fig. 3.

A continued fraction approach

We then look at a $depth = 1$ continued fraction of the form:

$$V_{1,2}(r) \approx a_1\,5^{-r} + a_2\,33^{-r} + \frac{a_3\,5^{-r} + a_4\,33^{-r}}{a_5\,5^{-r} + a_6\,33^{-r} + 1} \tag{12}$$

with $\{a_1, \ldots a_6\}$ all integers and a_5, a_6 both positive. A good approximation with $MSE \approx 6.45695718..$ was found with a single root in $r \approx 3.3757056527...$ with $V_{1,2}(r_{min}) \approx -98.0147$ for $r_{min} \approx 3.79036397045101....$ In this case, the values of the coefficients are $a_1 = -79105, a_2 = 46517936, a_3 = -7424260317, a_4 = -9999966733, a_5 = 3238247794$, and $a_6 = 4545$.

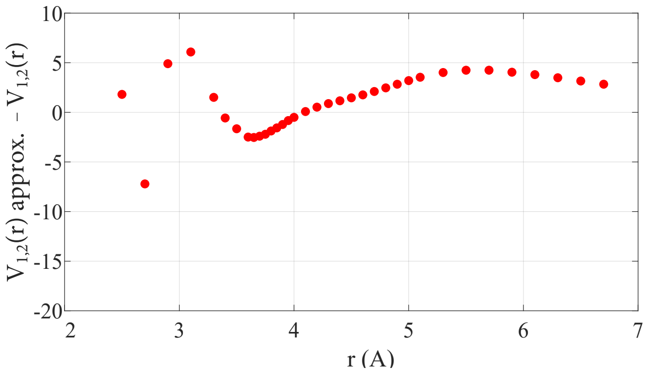

Figure 2. Error made for prediction ($V_{1,2}(r) \approx 46598041\,33^{-r} - 79879\,5^{-r}$) of the experimental ($V_{1,2}(r)$) Argon data by eq. (10)

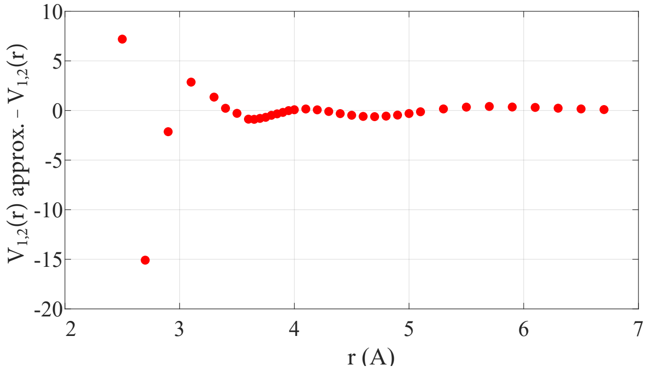

Figure 3. Error made for prediction ($V_{1,2}(r) \approx 47758692\,33^{-r} - 136107\,6^{-r} - 371\,2^{-r}$) of the experimental ($V_{1,2}(r)$) Argon data by eq. (11).

Another Continued Fraction Model with 3 Dirichlet Terms

We found a continued fraction model of $depth = 1$ which is using three Dirichlet terms as follows:

$$V_{1,2}(r) \approx \left(a_1\,30^{-r} + a_2\,7^{-r} + a_3\,2^{-r}\right) + \frac{(a_4\,30^{-r} + a_5\,7^{-r} + a_6\,2^{-r})}{(a_7\,30^{-r} + a_8\,7^{-r} + a_9\,2^{-r})} \tag{13}$$

with $\{a_1, \ldots a_9\}$ all integers and a_7, a_8, a_9 are positive. A good approximation with $MSE \approx 2.440429779..$ was found with a single root in $r \approx 3.37422355254799...$

In this case, the values of the coefficients are $a_1 = 40276481, a_2 = -270016, a_3 = -381, a_4 = 499980631, a_5 = 499999998, a_6 = -409990730, a_7 = 499939947, a_8 = 499999985$ and $a_9 = 414268318$. The plot of error is shown in Fig. 4.

We tabulated the approximation of $V_{1,2}(r)$ by the models in eqs. (8), (10), (11), (13) in Table. S1 with highlighting the best approximation in bold-face.

Appendix 2 - Comparison of error made for prediction of the experimental Argon data by eq. (11) and our SAAP prediction by eq. (1) (eq. (14) in paper) with parameter optimisation by min-based weighting

We used Mean Squared Error

$$MSE = \frac{(approx - exact)^2}{n},$$

Relative Error

$$RE = abs\left(\frac{(approx - exact)}{exact}\right)$$

Table S1. Approximations of the interatomic potential of the experimental Argon data of Halpern ([1]) by three models presented in this work. The best approximating value of the three equations is shown in boldface.

$r(A)$	$V_{1,2}(r)$	eq. (8)	eq. (10)	eq. (11)	eq. (13)
2.5	6018.01	6022.532	6019.821	6025.202	**6019.471**
2.7	2673.08	2660.961	2665.868	2657.992	**2667.46**
2.9	1083.88	**1085.532**	1088.785	1081.741	1087.747
3.1	363.94	369.3474	370.026	**366.7986**	368.2436
3.3	58.41	60.79563	59.92127	59.75802	**58.97633**
3.4	-14.92	-14.242	-15.4878	**-14.6985**	-15.8226
3.5	-58.41	**-58.6524**	-60.0644	-58.698	-59.8268
3.6	-81.69	-82.7545	-84.1818	**-82.5638**	-83.4839
3.65	-88.36	-89.498	-90.892	**-89.2512**	-90.0193
3.7	-92.56	-93.6227	-94.963	**-93.3576**	-93.956
3.75	-94.73	-95.6681	-96.9391	**-95.4195**	-95.8389
3.8	-95.39	-96.0774	-97.2677	**-95.877**	-96.1151
3.85	-94.75	-95.2145	-96.316	**-95.0903**	-95.1504
3.9	-93.16	-93.3766	-94.3843	-93.3531	**-93.2429**
3.95	-90.89	-90.806	-91.7173	**-90.9042**	-90.6349
4	-88.01	-87.6994	-88.5137	**-87.9369**	-87.522
4.1	-81.19	-80.4827	**-81.1079**	-81.0391	-80.3793
4.2	-73.63	-72.6557	-73.1057	**-73.5651**	-72.7262
4.3	-65.98	-64.8025	-65.0965	**-66.079**	-65.1256
4.4	-58.6	-57.2747	-57.4344	**-58.9161**	-57.9071
4.5	-51.78	-50.2692	-50.3168	**-52.2603**	-51.2466
4.6	-45.6	-43.8815	-43.8381	-46.1976	**-45.2212**
4.7	-40.13	-38.142	-38.027	-40.7518	**-39.8456**
4.8	-35.34	-33.0408	-32.8716	-35.9089	**-35.0974**
4.9	-31.17	-28.5443	-28.3357	-31.6329	**-30.9332**
5	-27.57	-24.6059	-24.3706	-27.8769	**-27.2996**
5.1	-24.46	-21.1736	-20.922	**-24.5893**	-24.1398
5.3	-19.37	-15.6158	-15.3551	**-19.2149**	-19.0212
5.5	-15.47	-11.4729	-11.2241	-15.1313	**-15.1759**
5.7	-12.43	-8.40766	-8.18221	-12.0249	**-12.2783**
5.9	-10	-6.15087	-5.95377	-9.65018	**-10.0791**
6.1	-8.13	-4.49468	-4.32683	-7.8213	**-8.39292**
6.3	-6.63	-3.2819	-3.1418	**-6.3998**	-7.08448
6.5	-5.44	-2.3951	-2.27999	**-5.28353**	-6.05585
6.7	-4.49	-1.74731	-1.65392	**-4.39747**	-5.2364
**MSE**		10.2459	9.0178	8.5568	2.4404

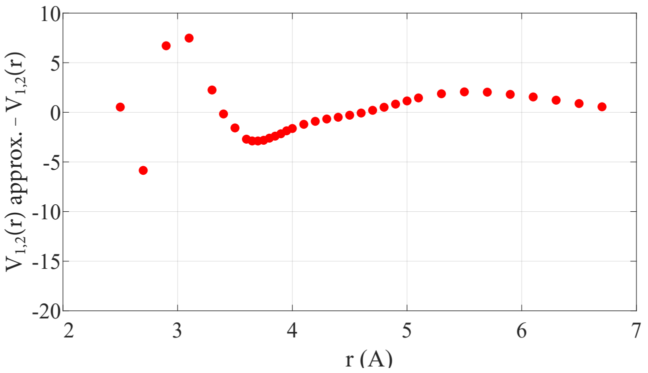

Figure 4. Error made for prediction ($V_{1,2}(r) \approx (a_1\, 30^{-r} + a_2\, 7^{-r} + a_3\, 2^{-r}) + \frac{(a_4\, 30^{-r} + a_5\, 7^{-r} + a_6\, 2^{-r})}{(a_7\, 30^{-r} + a_8\, 7^{-r} + a_9\, 2^{-r})}$) of the experimental ($V_{1,2}(r)$) Argon data by eq. (13).

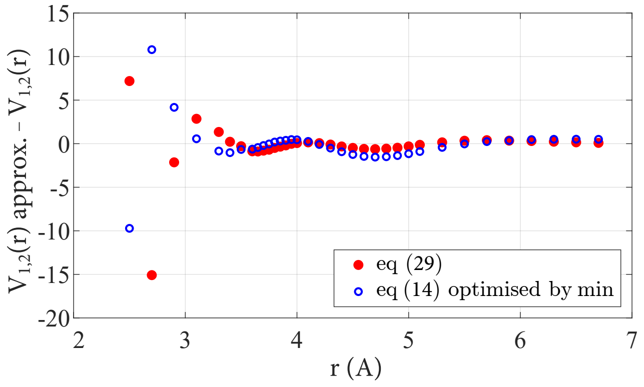

Figure 5. Comparison of error made for prediction of the experimental Argon data by eq. (11) and our SAAP prediction by eq. (1) with parameter optimisation by *min*-based weighting.

and min

$$min = \frac{(approx - exact)^2}{(exact - \lfloor exact_{min} \rfloor)^2}$$

based weighting in the fitness function for optimising the parameters value. We found the value of parameters $\{a_0, a_1, a_2, a_3, a_4, a_5\}$ in eq. (1) as: {1642849.852, -1.324763297, -21325.92292, -0.56953138, -2453.696838, 0.007041875} for *MSE*, {1518906.938, -1.289763354, -19413.1251, -0.486655272, -2034.237159, 0.007105139} for *RE* and {1513858.734, -1.292491738, -17238.23531, -0.419519278, -1545.992086, 0.00704256} for *min* based weighting. The approximations of the SAAP for these parameter values are shown in Table. S2.

We presented the comparison of the error made by our best approximation for $V_{1,2}(r)$ in eq. (11) and the SAAP approximation by eq. (1) for the optimisation of parameter with *min* in Fig. 5.

Appendix 3 - Numerical and representational considerations

On the use of integer coefficients

While the definition of our base representations, starting from the use of the general Dirichlet series that motivated our study, does not require coefficients to be integers, we nevertheless restricted our equations to have integers only. One reason is pretty obvious, given in this way, all our approximations are "unambiguous" because they do not rely on truncating coefficients derived from a floating-point representation. The other reason is perhaps not so obvious so it is proper to discuss it here.

Since $n_1^{-r} n_2^{-r} = (n_1\, n_2)^{-r}$, an application of the Dirichlet series is as a generating series for counting weighted sets of objects with respect to a weight that is combined multiplicatively when taking Cartesian products. However, this equality has importance for the development of data-driven symbolic regression models when the coefficients are restricted to be integers. As we have said before, a symbolic regression method that allows the multiplication of variables, would allow the presence

of a term proportional to $n_1^{-r} n_2^{-r}$ in an equation. If another equation is generated during the run, using $(n_1\, n_2)^{-r}$ instead, all other terms equal, this one might have lower complexity and the heuristic would probably prefer it (since many symbolic regression algorithms are biased towards selecting lower complexity formulas). Consequently, this has also the desired effect that the coefficients present in that equation will be further optimised during the run. In our experience, this is far from being something of a negligible effect, and we have preferred to use integer coefficients when evolving formulas with TuringBot instead of employing the floating-point arithmetic functionality (which tends to produce formulas that diverge a bit on their fitting performance but are much more functionally similar between them).

Finally, having produced simple equations only defined by a few integers such as those for Helium ((2), (3)) which present a great fit to the given data have a certain "beauty" associated with them. We can not say anything at this stage about why is that the case, but it is obvious that further non-linear optimisation and replacement of these integers by rationals is the next possible step. We thought at this stage that leaving these approximations with integers coefficients somehow highlights the potential of exploring the Dirichlet series representation.

On the opportunities for parallelization

It may have not escaped to the reader that eq. (5) is composed of three-factor terms thus it presents itself as something that can be concurrently computed. eq. (5) was obtained, thanks to a symbolic computation program (Mathematica,) followed by further refining of the coefficients to obtain integer values. In this section, we illustrate that perhaps for other more complex potentials, there is also a way to use parallelism to compute continued fractions of higher depth. This said, there is another way to prepare a code for parallelism of these computations thanks to combining two results.

$$\frac{p_n}{q_n} = a_1 + \cfrac{b_2}{a_2 + \cfrac{b_3}{a_3 + \cfrac{b_4}{a_4 + \ddots + \cfrac{b_n}{a_n}}}} \tag{14}$$

Milne-Thomson introduced matrix representation of continued fractions[2] that gives an extra opportunity for parallelism[3]. Let C_n be a matrix that contains the numerators and the denominators of the convergents of order n and $n-1$, i.e. (p_n/q_n) and (p_{n-1}/q_{n-1}) respectively; then C_n is defined as:

$$C_n = \begin{bmatrix} p_n & p_{n-1} \\ q_n & q_{n-1} \end{bmatrix} \tag{15}$$

C_n can be written as:

$$C_n = A_1 \times A_2 \times A_3 \times \cdots \times A_n \tag{16}$$

with

$$A_k = \begin{bmatrix} a_k & 1 \\ b_k & 0 \end{bmatrix} \tag{17}$$

for $1 \leq k \leq n$, and $b_1 = 0$. For $k = 3$ we have:

$$\begin{bmatrix} p_n & p_{n-1} \\ q_n & q_{n-1} \end{bmatrix} = \begin{bmatrix} a_1 & 1 \\ 1 & 0 \end{bmatrix} \times \begin{bmatrix} a_2 & 1 \\ b_2 & 0 \end{bmatrix} \times \begin{bmatrix} a_3 & 1 \\ b_3 & 0 \end{bmatrix} \tag{18}$$

Then, for eq. (4), we only need to compute r^{-6} and 33^{-r} once for each pair and then we have, $a_1(r) = 0$, $b_1 = 1$, $b_2 = 29173.2876433231$, $a_2 = 14.2052906553669\ 31^{-r} + 0.348000451488318$, $b_3 = 0.000325594052656555$ and $a_3 = 31^{-r} - 0.000114634476140062$.

On the challenges for optimisation methods

Contrary to what the relatively small size of the instance of this problem may suggest, the optimisation problem associated to fitting these functional forms is far from being trivial. It is timely to recall John von Neumann's quote: *"With four parameters I can fit an elephant, and with five I can make him wiggle his trunk"*[4] which has led to some interesting and imaginative, and sometimes very clever tongue-in-cheek, contributions[5,6]. This said, in a dataset such as that of Helium, and we are appealing to the imagination of the reader here, if we see in Fig. 2 of the paper, the silhouette of a head and a raised trunk of an elephant, and if we normalise these values such that the difference between the potential range (-12 and 4) would correspond to one meter, then the raised up "trunk" of the elephant would be nearly 18 kilometres long (indeed, the highest ab initio value to fit for Helium is 286570 for $r = 0.529177$).

We doubt von Neumann was thinking in such unusual elephants, but we take his word of warning that too many parameters would likely produce a great approximation. Still, in this dataset the need to having a very good fit nearly the global minimum, as well as having the right asymptotic behaviour, obliges us to find a way to deal with this range in values. This is essential for all the important thermodynamical observables that are derived from simulations that use these approximations. SAAPx requires seven rational numbers (i.e. 14 integers), one integer (1) and one transcendental (e), while eqs. (2) and (3) are only defined by six integers, and eq. (5) by nine integers. After having experimented with these datasets for a while, we think it will be perhaps possible but really challenging to find good approximations which are only defined by six or five integers and that have the expected asymptotic behaviour outside the interval range of distances on which ab initio experimental data exists. In fact, we have been very close to obtain

On the integer sequence of Moser's circle problem

For readers curious about the nature of the sequence used to illustrate the computation of solutions by symbolic and continued fraction regression, we will explain its origin here. The first terms may induce a reader to think that we are in the presence of the infinite sequence $a_n = 2^{(n-1)}$ with $\{1, 2, 4, 8, 16, 32, ...\}$. Since the similarity soon breaks this subsequence can be seen as an example of the problem of extrapolating in numerical regression beyond the range of values of the training set.

Assume that you have a circle and r points are located in the inscribing circumference. Let's now draw all the segments that connect all points. If we add the extra restriction that the r positions are such that no three lines intersect at the same point then the value of $f(r)$ corresponds to the number of sections that a circle is divided. The problem is also known as *Moser's circle problem* (`https://mathworld.wolfram.com/CircleDivisionbyChords.html`). A nice video on the topic is available online thanks to `3Blue1Brown` (`https://www.youtube.com/watch?v=K8P8uFahAgc`). More terms of the sequence, and other formulas, can be found at the Online Encyclopedia of Integer Sequences (A000127) (`https://oeis.org/A000127`). In fact, eq. (7) is indeed the true unknown function that represents the solutions of Moser's circle problem for an arbitrary number of r points. This means that the generalisation for values of r out of the domain is then correct in this case.

References

1. Halpern, A. M. Structural and thermodynamic properties of the argon dimer. *J. Chem. Educ.* **87**, 174–179, DOI: 10.1021/ed800049s (2010). https://doi.org/10.1021/ed800049s.

2. Milne-Thomson, L. M. A matrix representation of ascending and descending continued fractions. *Proc. Edinb. Math. Soc.* **3**, 189–200, DOI: 10.1017/S0013091500007926 (1933).

3. Eğecio glu, O., Koç, Ç. K. & Coma, J. R. Fast computation of continued fractions. *Comput. & Math. with Appl.* **21**, 167–169, DOI: https://doi.org/10.1016/0898-1221(91)90095-L (1991).

4. Dyson, F. A meeting with Enrico Fermi. *Nature* **427**, 297 (2004).

5. Mayer, J., Khairy, K. & Howard, J. Drawing an elephant with four complex parameters. *Am. J. Phys.* **78**, 648–649, DOI: 10.1119/1.3254017 (2010). https://doi.org/10.1119/1.3254017.

6. Boué, L. Real numbers, data science and chaos: How to fit any dataset with a single parameter, DOI: 10.48550/ARXIV.1904.12320 (2019).

Table S2. Table for the exact ($V_{1,2}(r)$) and the $SAAP(r)$ approximations of $V_{1,2}(r)$ provided by eq. (1) for r in the range specified in the Halpern's dataset for Argon. We investigated in this case the optimisation of the parameters using different weighting: *MSE, RE, min* in the fitness function. The best prediction value by models is shown in boldface.

$r(A)$	$V_{1,2}(r)$	$V_{1,2}(r) \approx SAAP(r)$ approx. by eq. (1)		
		(with MSE)	(with RE)	(with min)
2.5	6018.01	**6017.089**	5991.089	6008.293
2.7	2673.08	2676.574	**2675.038**	2683.877
2.9	1083.88	1081.479	**1084.879**	1088.053
3.1	363.94	361.543	**364.2321**	364.5062
3.3	58.41	57.23909	**58.41**	57.56429
3.4	-14.92	-15.4947	**-14.9266**	-15.9483
3.5	-58.41	**-58.1417**	-58.0227	-59.0723
3.6	-81.69	-81.1416	**-81.3281**	-82.3129
3.65	-88.36	-87.5739	-87.8661	**-88.7968**
3.7	-92.56	-91.531	-91.9021	**-92.769**
3.75	-94.73	-93.5363	-93.9628	**-94.7596**
3.8	-95.39	-94.0162	-94.4778	**-95.2006**
3.85	-94.75	-93.3168	-93.7963	**-94.443**
3.9	-93.16	-91.7186	-92.2014	**-92.7717**
3.95	-90.89	-89.4479	-89.9221	**-90.4168**
4	-88.01	-86.6869	-87.1428	**-87.5637**
4.1	-81.19	-80.2478	-80.6457	**-80.9272**
4.2	-73.63	-73.2445	**-73.5666**	-73.7223
4.3	-65.98	**-66.199**	-66.4371	-66.482
4.4	-58.6	**-59.4211**	-59.5737	-59.5233
4.5	-51.78	-53.0822	-53.1524	**-53.0216**
4.6	-45.6	-47.2647	-47.2585	**-47.0612**
4.7	-40.13	-41.995	-41.9203	**-41.6687**
4.8	-35.34	-37.2653	-37.1305	**-36.8359**
4.9	-31.17	-33.048	-32.8618	**-32.5338**
5	-27.57	-29.305	-29.0759	**-28.7229**
5.1	-24.46	-25.9939	-25.7296	**-25.3589**
5.3	-19.37	-20.4946	-20.1812	**-19.7921**
5.5	-15.47	-16.2266	-15.8871	**-15.4967**
5.7	-12.43	-12.9156	**-12.5673**	-12.187
5.9	-10	-10.3413	**-9.99653**	-9.63375
6.1	-8.13	-8.33216	**-7.99934**	-7.6583
6.3	-6.63	**-6.75647**	-6.44095	-6.12381
6.5	-5.44	**-5.5139**	-5.21876	-4.92625
6.7	-4.49	**-4.52825**	-4.25495	-3.98682
